# Extraction and Valorization of Oilseed Cakes for Value-Added Food Components—A Review for a Sustainable Foodstuff Production in a Case Process Approach

**DOI:** 10.3390/foods14132244

**Published:** 2025-06-25

**Authors:** Nada Grahovac, Milica Aleksić, Biljana Trajkovska, Ana Marjanović Jeromela, Gjore Nakov

**Affiliations:** 1 Institute of Field and Vegetable Crops, Maksima Gorkog 30, 21000 Novi Sad, Serbia or aleksic.96.17.b@uns.ac.rs (M.A.); ana.jeromela@ifvcns.ns.ac.rs (A.M.J.); 2 Faculty of Technology Novi Sad, University of Novi Sad, Bulevar cara Lazara 1, 21000 Novi Sad, Serbia; 3 Faculty of Biotechnical Sciences—Bitola, University “St. Kliment Ohridski”—Bitola, 7000 Bitola, North Macedonia; biljana.trajkovska@uklo.edu.mk; 4 College of Sliven, Technical University of Sofia, 8800 Sliven, Bulgaria; gnakov@tu-sofia.bg

**Keywords:** oilseed cakes, protein extraction, bioactive compounds, sustainable valorization, circular economy, functional foods

## Abstract

Oilseed cakes, by-products of oil extraction, represent an underutilized resource with significant potential for sustainable food and pharmaceutical applications. This comprehensive review examines the valorization strategies for oilseed cakes, focusing on their rich protein (up to 56%) and fiber (up to 66%) content. We analyze both conventional and innovative extraction methods, highlighting the advantages of ultrasound-assisted (96.64% phenolic compound yield), enzymatic (82–83% protein recovery), and subcritical water extraction techniques in improving efficiency while reducing environmental impact. This review demonstrates diverse applications of oilseed cake components from gluten-free bakery products and plant-based meat alternatives to advanced nanoencapsulation systems for bioactive compounds. Each major oilseed type (soybean, rapeseed, sunflower and flaxseed) exhibits unique nutritional and functional properties that can be optimized through appropriate processing. Despite technological advances, challenges remain in scaling extraction methods and balancing yield with functionality. This paper identifies key research directions, including the development of integrated biorefinery approaches and the further exploration of health-promoting peptides and fibers. By addressing these challenges, oilseed cakes can play a crucial role in sustainable food systems and the circular economy, transforming agricultural by-products into high-value ingredients while reducing waste.

## 1. Introduction

Oilseed cakes are by-products obtained after oil extraction from seeds, with their composition varying depending on the extraction method (e.g., mechanical pressing, solvent extraction, cold pressing, or supercritical CO_2_ extraction) [1,2]. These cakes are generally classified as edible or non-edible. Edible types are rich in proteins, fibers, minerals, and bioactive compounds, while non-edible varieties often contain antinutritional or toxic substances. For instance, non-edible oilseed cakes, such as those derived from jatropha, karanja, mahua, and castor, contain various toxic compounds—curcin in jatropha, ricin in castor, saponins and tannins in mahua, and karanjin, pongamol, and glabrin in karanja. Due to their toxicity, these cakes are unsuitable for food and feed applications and are instead utilized in agriculture as fertilizers or as feedstock for biogas and biofuel production [1,3,4,5]. Given the growing global population, climate change, and resource depletion, there is an urgent need for sustainable food production practices [6,7]. At the same time, food loss and waste remain critical global challenges; approximately one-third of the world’s food is wasted, and oilseeds account for about 20% of this waste [8]. In this context, oilseed cakes represent an underexploited agricultural by-product with the potential to enhance the efficiency and circularity of agri-food systems. Researchers and industries are increasingly embracing circular economy principles, aiming to valorize oilseed cakes—traditionally used as animal feed or fertilizer—into high-value ingredients for the food and pharmaceutical industries [6]. These cakes retain approximately 40–50% of the seed’s original proteins, fibers, minerals, and bioactive compounds [4], making them promising sources of functional ingredients such as antioxidants, enzymes, and protein isolates. Such components not only contribute to nutritional improvement but also offer significant economic value in the context of waste valorization, with recovery rates depending on the extraction method employed [9,10]. The rising demand for health-promoting food ingredients has driven the growth of the functional food market since the 1990s, positioning oilseed cakes as promising sources of protein, fiber, and phenolic compounds [11]. Their high nutritional composition, low cost, and year-round availability further support their integration into sustainable biotechnological processes and value-added food chains.

Despite these advantages, the full potential of oilseed remains underutilized, primarily due to technological limitations and scalability issues in the extraction and refinement of their valuable compounds. Accordingly, recent research has increasingly focused on developing efficient, scalable, and environmentally friendly extraction technologies suited for industrial application.

This review aims to highlight the importance of oilseed cakes as a sustainable resource and to provide a comprehensive overview of their composition, functional potential, and applicability in food, pharmaceutical, and biotechnology sectors. Particular attention is given to comparing conventional and innovative extraction methods, including their efficiency, environmental impact, and industrial scalability. The overarching aim is to identify sustainable and cost-effective solutions that bridge the gap between laboratory research and industrial implementation, contributing to the advancement of circular and zero-waste food production systems.

## 2. Oilseeds and Oilseed Cakes: Composition and Global Significance

### 2.1. Nutritional and Functional Profiles of Major Oilseed Crops

Edible oilseed cakes represent valuable by-products of oil extraction, characterized by their high protein content and variable nutritional composition, which is influenced by genetic, agronomic, and processing factors [6,12]. Understanding their nutritional and functional profiles is crucial for advancing valorization strategies. This section outlines the proximate composition, amino acid profiles, and micronutrient content of the major oilseed crops—sunflower, soybean, rapeseed, and flaxseed and their corresponding cakes. As detailed in Table 1, significant variations exist in macronutrient content across species. Edible oilseed cakes can contain up to 56% protein, 66% fibers, and a wide range of micronutrients such as minerals, phenolic compounds, and vitamins. In contrast, non-edible varieties often contain antinutritional compounds, which limit their use to non-food applications such as fertilizers [1,3,4,5,13]. For instance, canola cake is rich in sinapic acid derivatives with antioxidant properties, while sunflower cake is notable for its content of chlorogenic acids [14,15]. These bioactive components, along with complete amino acid and micronutrient profiles, are detailed in Table 2 and Table 3.

Sunflower seeds typically contain 28–60% oil, 10–25.5% protein, and 16–55% fiber (Table 1). Among amino acids, glutamic and aspartic acids predominate (Table 2), and sunflower contains relatively low levels of antinutritional factors and protease inhibitors compared to other oilseeds (Table 3).

After oil extraction, sunflower cake retains 1–23.6% residual oil, 29–43.4% protein, and 13–36% fiber, highlighting its nutritional potential.

Soybean seeds exhibit an oil content ranging from 7.3% to 24.7% oil, protein from 14% to 47%, and fiber from 5.6% to 31.9% (Table 1). The functional properties of soybean proteins, as reflected in the proximate analysis, make them indispensable in food processing [28]. Soybeans are particularly rich in lysine, an essential amino acid (Table 2), and contain beneficial compounds such as isoflavones, although moderate levels of antinutritional factors are also present (Table 3). Soybean cake contains 0.6–8.9% residual oil, 25.7–52.4% protein, and 3–6% fiber, making it one of the most widely used protein-rich feed resources.

Rapeseed demonstrates a balanced nutritional profile, with an oil content between 31.2% and 47.5%, protein content of 18.6–20.1%, and fiber content around 12.8% (Table 1). Although its amino acid profile is relatively complete, it is deficient in methionine (Table 2), and its nutritional utility is reduced by glucosinolates, which are considered antinutritional compounds (Table 3). The rapeseed cake composition varies depending on processing and seed type, with the residual oil content ranging from 1.1% to 31.3%, protein from 14% to 45%, and fiber from 5.5% to 19.5%.

Flaxseed is especially valued for its high content of functional and health-promoting compounds. Its oil content ranges from 30% to 42.2%, protein between 18.3% and 30%, and fiber from 20% to 35% (Table 1). It is particularly rich in arginine (Table 2) and contains lignans antioxidants associated with various health benefits (Table 3). The resulting flaxseed cake retains 7–21.4% oil, 36–56% protein, and approximately 66% fiber, confirming its suitability for use in functional foods.

#### Global Production Trends and Market Importance

The global oilseed market is primary dominated by four major crops, each offering distinct compositional profiles attributes and industrial applications. Sunflower ranks as the third most cultivated oilseed globally, with a projected production of 57.5 million tons in the 2023/2024 season, making a 5.2% year-on-year increase [16]. It is widely appreciated for its adaptability to a broad range of climatic and soil conditions. Soybean, expected to reach 393.4 million tons (4% increase), remains the nutritional standard among oilseeds due to its exceptionally high protein content and widespread use in both human nutrition and animal feed [20]. Rapeseed, with a forecasted output of 89.1 million tons (2023/2024), shows a slight decline of 1.4% compared to the previous season, yet continues to play a vital role in the global oilseed market [24]. Although flaxseed is produced in relatively lower quantities, it adds unique nutritional value to the oilseed portfolio through its richness in omega-3 fatty acids and lignans.

**Figure 1:**
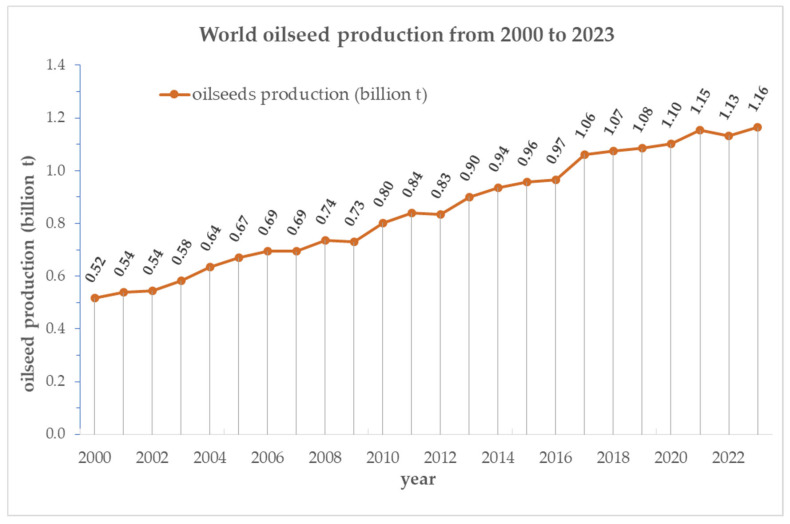
Global oilseed production trends from 2000 to 2023 (in billion tons).

Global oilseed production continues to expand, reaching 1.132 billion tons in 2022 and 1.163 billion tons in 2023. Correspondingly, oilseed cake production increased from 427.249 million tons to 446.568 million tons over the same period [8]. These figures reflect a sustained upward trend in oilseed output, which has more than doubled over the last two decades—from 0.52 billion tons in 2000 to 1.16 billion tons in 2023 (Figure 1). This continued growing is driven by the increasing global demand for vegetable oils and plant-based proteins as well as the strategic role of oilseed crops in advancing sustainable food systems and bioeconomy strategies.

## 3. Current and Emerging Applications of Oilseed Cakes

Building upon the nutritional composition outlined in the previous section, oilseed cakes serve multiple applications that capitalize on their protein, fiber, and micronutrient content. While their traditional uses as components of animal feed, organic fertilizers, and biomass fuel remain prominent, innovative applications are emerging to address both nutritional and environmental challenges. In the food sector, oilseed cakes are being used in the development of functional products such as gluten-free bakery items (e.g., bread and cookies) and plant-based meat alternatives like vegan burgers [56,57]. Additionally, compressed dietary supplements made from sunflower, coconut, pumpkin, and flaxseed cakes have also been developed for direct human consumption [58]. Beyond food, non-edible oilseed cakes are gaining attention in biotechnological applications. They serve as valuable substrates for the production of lactic acid, enzymes, biofuels, and biocontrol agents, and are also being explored for use in soil enrichment and mushroom cultivation [1].

The high protein and fiber content documented in Table 1 makes oilseed cakes valuable feed ingredients for omnivores, ruminants, poultry, pigs, and aquatic animals [6]. However, as identified in Table 3, the presence of antinutrients like glucosinolates, phytates, and oxalates limits their universal adoption. Rapeseed cake exemplifies this paradox; while Table 2 confirms its balanced amino acid profile, its utilization remains restricted to ruminants due to the antinutrients detailed in Table 3 [24,48]. Processing techniques, including fermentation, thermal treatment, and alkaline extraction, can mitigate these limitations by improving protein digestibility and reducing antinutritional factors [59,60]. The mineral composition highlighted in Table 3 supports the growing use of oilseed cakes as organic fertilizers. Their rich content of calcium, magnesium, phosphorus, and micronutrients [26] aligns with sustainable soil management practices. This application represents a circular approach to utilize the nutritional components characterized in the previous section. The carbohydrate and lignin fractions identified in Table 1 enable thermochemical conversion into bio-oil through pyrolysis [26]. Additionally, the fermentable sugars in certain cakes support bioethanol production [61]. These applications address the environmental concerns associated with cake decomposition while creating value from the compositional elements detailed earlier.

The same bioactive compounds that present antinutritional challenges in feed applications (Table 3) become valuable substrates for biotechnological processes. Oilseed cakes serve as raw materials for producing the following:

- Enzymes (lipase, phytase, peptidase, and α-amylase), leveraging their protein profiles (Table 2);
- Biopesticides, utilizing their phenolic compounds;
- Protein hydrolysates, capitalizing on their amino acid composition;
- Bioactive peptide preparations [1].

The transition from traditional to innovative uses reflects an evolving understanding of oilseed cake composition. As processing technologies advance, the nutritional constraints identified in the previous section become opportunities for value-added applications. Particularly promising approaches are integrated biorefinery approaches that simultaneously address the protein, fiber, and micronutrient fractions characterized (Table 1, Table 2 and Table 3).

## 4. Extraction Techniques for Value-Added Compounds from Oilseed Cakes

### 4.1. Protein Recovery from Oilseed Cakes: Challenges and Opportunities

With the continuous growth of the global population, the demand for sustainable and affordable sources of food and protein is increasing. Conventional extraction methods such as alkaline or acidic solubilization and Soxhlet extraction are often solvent-intensive and environmentally detrimental [6,62,63,64]. In contrast, enzyme-assisted extraction can reduce energy consumption by up to 40%, and novel techniques like ultrasound-assisted, subcritical water, and deep eutectic solvents provide greener and more efficient alternatives. These methods enable the selective recovery of proteins and polyphenols, preserving their bioactivity and functional properties [63,65]. As a result, the food industry is turning to alternative sources, such as by-products from the oilseed industry. Oilseed cakes are protein-rich residues that can serve as cost-effective sources of plant-based proteins to enhance the nutritional value of food. Plant proteins are often embedded within cell walls or intracellular matrices, making cell disruption essential for effective extraction [66]. Understanding the structural organization of proteins and associated components is crucial for designing efficient extraction methods [67]. The aim is to achieve high yields with minimal purification steps and costs, while preserving nutritional and functional properties and eliminating antinutrients [19]. Pretreatment steps like dehulling and milling improve extraction efficiency by removing polyphenols and increasing the accessibility of protein bodies. Mechanical pressing not only extracts oil but also facilitates protein release by damaging plant cells [67].

#### 4.1.1. Conventional Protein Extraction Methods

Aqueous extraction, which solubilizes proteins using alkali or acid solutions followed by centrifugation, remains the most common traditional method. Acidic extraction (pH 2) yields 30–50% protein, while alkaline extraction (pH 10) yields up to 90% [19]. Adding NaCl (0–0.5 mol/L) at pH 6 improves the yield from 40% to 60%. A higher pH increases yields (e.g., pea 49.20% to 57.56% at pH 8.5–9.5 [56]; pigeon pea 35.1% to 58.1%; and chickpeas 36.4% to 53.5% at pH 8.5–12.5 [68]). However, strong alkaline conditions can denature proteins via exposure of hydrophobic and sulfhydryl groups, causing unfolding and polymerization [19,66,69]. Higher temperatures often increase the yield of extracted protein (e.g., extraction of protein from hempseed cake at room temperature had a yield of 35%, while extraction at 50 °C resulted in yield of 55%) [70], but often cause protein denaturation and a loss of functional properties [68]. The extraction time typically ranges from 10 min to 1 h; extending the extraction time to 2 or 3 h did not result in a significant improvement in yield [68,70]. Defatted material gives a better yield (40–50%) than full-fat materials [19]. For example, canola protein isolates extracted under alkaline conditions show poor solubility/function [71], while flax and sesame perform well [72]. In the case of sunflower, phenolic compounds should be removed by organic solvents before extraction to avoid quality loss [6,73]

#### 4.1.2. Emerging Technologies for Enhanced Protein Extraction

##### Ultrasonic-Assisted Extraction: Efficiency and Optimization

Ultrasonic extraction is an environmentally friendly and efficient method used alone or as a pretreatment, reducing time, energy, and solvent use, with up to 20% higher protein yields than conventional methods. However, ultrasound may alter protein structures, causing denaturation and functional changes [66]. Optimal conditions vary: for rapeseed protein, 5.6 W/cm^3^ at 45 °C; for sunflower, 220 W/L (watts per liter) at 45 °C for 15 min. Temperatures above 45 °C do not improve the yield and may harm protein functionality [33,74].

##### Enzymatic Hydrolysis for Improved Protein Yield

Enzymatic-assisted extraction effectively improves the protein yield and functionality by using enzymes (carbohydrases, pectinases, proteases) to degrade cell walls and disrupt protein bonds [66,75,76]. Studies report yields of 82–83% using enzyme mixtures on oilseed cakes [77,78]. Although this method requires longer processing times compared to conventional acid or alkaline extraction, higher costs, and precise control of pH and temperature, it operates under milder, more environmentally friendly conditions, yielding nutritionally superior proteins [79,80].

##### Microwave-Assisted Extraction: Mechanisms and Applications

Microwave-assisted extraction utilizes electromagnetic radiation (300 MHz–300 GHz) to generate heat that disrupts hydrogen bonds and improves solvent penetration, making it effective for tough plant materials resistant to other methods [66,76]. This environmentally friendly technique has shown promising protein yields: 58% in soybeans (2450 MHz, 60 °C, 30 min) [81], a 24% increase in soymilk protein extraction [82], and a 33% higher yield in rice grains compared to alkaline extraction, with improved functional properties like foaming and emulsification [83].

##### Pulsed Electric Field (PEF) Extraction: A Non-Thermal Alternative

PEF extraction is an emerging non-thermal technique that applies short, high-voltage pulses (ranging from 100–300 V/cm to 10–50 kV/cm) to disrupt cell membranes and release proteins with minimal denaturation. In addition to extraction, it is used for food preservation and microbial inactivation [80]. However, compared to other innovative methods, PEF extraction is generally less efficient for protein recovery [66,84]. Studies have explored its application across various plant matrices. For instance, the PEF pretreatment of rapeseed biomass significantly increased the protein yield compared to untreated samples [85] and enhanced the recovery of proteins and polyphenols from sesame cake compared to conventional methods [86].

##### High-Pressure and Hydrostatic Extraction Techniques

High-pressure extraction (HPE) techniques, using liquids or gases, and high hydrostatic pressure (HHP), using water, apply 100–1000 MPa to disrupt cell walls and improve solvent penetration, preserving protein functionality [66]. The efficiency depends on the protein type, medium, pressure, pH, and ionic strength. For instance, increasing the pressure from 50 MPa to 100 MPa led to a rise in protein yield from 88% to 95% for the okara solution [80,87]. In flax seeds, a 300 MPa treatment yielded 86,1% protein while also increasing peptide antioxidant activity [88]. Similarly, pressures of 500 MPa and 200 MPa enhanced protein recovery in rice bran (66.3%) and soybean flakes (10.9% increase over conventional methods), respectively [89,90].

### 4.2. Recovery of Bioactive Polyphenols from Oilseed By-Products

Oilseed processing by-products contain valuable bioactive components with antimicrobial, antioxidative, and anti-inflammatory properties, widely used in food, pharmaceutical, and cosmetic industries [91,92]. The choice of extraction method depends on the raw material, compound characteristics, efficiency, cost, environmental impact, and scalability. Oilseed cakes are rich in phenolics—including free, esterified, and condensed acids, flavonoids, and lignins—exhibiting antioxidant, anticancer, antiteratogenic, and antimicrobial effects, with profiles varying by seed type (see Table 4). Extraction typically employs polar solvents (ethanol, methanol, ethyl acetate) or solvent–water mixtures to enhance the yield. Environmentally friendly solvents like deep eutectic solvents and supercritical fluids are gaining popularity. Advanced methods such as ultrasonic, microwave, pulsed electric field, and supercritical fluid extraction increasingly replace conventional techniques for improved efficiency.

#### 4.2.1. Conventional Methods for Polyphenol Extraction

Conventional solid–liquid extraction techniques such as maceration, infusion, digestion, and Soxhlet extraction are suitable for thermolabile compounds and require minimal equipment. However, they involve large volumes of non-reusable organic solvents, long processing times, and multiple extraction cycles [97]. For example, ultrasonic extraction (water, 2 min, 20 kHz) outperformed traditional ethanol extraction (75% ethanol, 20 min, room temperature) for rapeseed phenols, yielding 7.93% more phenolic compounds [98].

#### 4.2.2. Advanced Techniques for Polyphenol Isolation

##### Ultrasonic-Assisted Polyphenol Extraction

Ultrasound enhances mass transfer through cavitation effects, where bubble formation and collapse disrupt cell structures and improve solvent penetration. Optimizing amplitude, frequency, and power is key for yield and selectivity [57,69]. For rapeseed phenols, optimal ultrasound conditions (amplitude 10, 8 min, water) reached a 96.64% yield. The methanol extraction of sunflower chlorogenic acid using ultrasound gave a 15% higher yield in 30 min compared to 1 h by conventional stirring [99]. Despite its efficiency, industrial adoption remains limited.

##### Supercritical Fluid and Pressurized Liquid Extraction of Phenolic Compounds

Pressurized liquid extraction (PLE) applies high temperatures (50–200 °C) and pressures to enhance solvent penetration and compound solubility, offering higher yields with less solvent but lower selectivity, often requiring further purification [100,101]. Rapeseed phenol extraction at 200 °C for 20 min (100 bar, 60% methanol) yielded 300% more than Soxhlet extraction (12 h, 100 °C) [102]. Supercritical fluids like CO_2_ extraction also show promise for phenolics in pumpkin, flax, and hemp seeds, with advantages in solvent removal and recycling [103]. These advanced extraction techniques, however, demand high initial costs and specialized technological skills, factors that should be considered for their industrial application.

##### Pulsed Electric Field (PEF) for Polyphenol Recovery

PEF technology allows for the cold extraction of heat-sensitive compounds via cell membrane electroporation. While effective for fruits and vegetables, its use in oilseeds needs further research [86]. Studies show that PEF extraction outperforms conventional methods but is less efficient than ultrasound and High-Voltage Electric Discharge (HVED) methods. For example, in olive kernels, PEF extraction was less effective than ultrasound or HVED [104], whereas in sesame cake, PEF extraction improved the phenol and protein recovery compared to conventional ethanol extraction [86]. Despite advantages like reduced solvent use and mild conditions, high equipment and energy costs currently limit industrial implementation [89].

### 4.3. Extraction and Processing of Dietary Fiber from Oilseed By-Products

Dietary fibers represent valuable by-products of plant material processing, remaining after the extraction of oils, juices, or other primary products. Approximately one-third of processed plant materials end up as waste, creating significant opportunities for valorization. While not directly hazardous to the environment, these fiber-rich residues can release unpleasant odors and harmful gases during decomposition, making their utilization important for environmental protection [30,105]. These polysaccharides, including cellulose, hemicellulose, lignin, and pectin, form the structural components of plant cell walls. Classified by solubility, fibers fall into water-soluble (pectins, β-glucan, inulin, gums) and insoluble (cellulose, hemicellulose, lignin) categories. Though resistant to human digestive enzymes, they play crucial roles in gastrointestinal function and offer various applications across food, pharmaceutical, and textile industries. The extracted fibers serve as functional food components, pharmaceutical supplements, or additives enhancing food properties [106].

#### 4.3.1. Traditional Fiber Extraction Methods

##### Dry Processing Techniques

Widely used in industrial settings, this method involves grinding seeds and separating components by density using air currents. While efficient and chemical-free, it produces an incomplete separation, making it primarily suitable for animal feed production. The process yields two fractions: lighter starch/fiber particles and heavier protein components [30,105].

##### Wet Processing: Acidic and Alkaline Approaches

These methods employ aqueous solutions, either acidic or alkaline, for a more efficient fiber separation. The traditional approach uses sulfuric acid, requiring up to 36 h and posing environmental concerns due to SO_2_ emissions. Modern adaptations utilize sodium hydroxide (NaOH) solutions at elevated temperatures, often combined with enzymes like proteases and α-amylases to accelerate cell disintegration. While more effective than dry methods, these processes still face challenges regarding time efficiency and environmental impact [105].

##### Gravimetric and Enzymatic Methods

These techniques include the following [105]:

- Non-enzymatic methods (acid-detergent and neutral-detergent extractions), which suffer from fiber loss or incompatibility with soluble fiber-rich materials;
- Enzymatic methods that use α-amylase, protease, and amyloglucosidase to break down starch and proteins before ethanol precipitation.

Despite their effectiveness, gravimetric methods require significant solvent and energy inputs. Reducing ethanol concentration from 76% to 41–56% can improve environmental sustainability [105,107].

##### Chemical Extraction: Challenges and Limitations

Traditional chemical approaches involve concentrated sulfuric acid digestion followed by sequential filtration with sodium hydroxide and ethanol washing. While effective, these methods pose worker safety concerns and environmental risks due to their use of hazardous chemicals [105].

#### 4.3.2. Innovative Fiber Extraction Technologies

##### Microwave Extraction Assisted Fiber Extraction

Operating at 300 MHz to 300 GHz, this method rapidly heats solvents and samples, disrupting cellular structures for improved mass transfer. While offering reduced solvent use and higher yields, microwave extraction faces challenges in industrial scaling and high energy demands. The rapid heating can modify fiber structures, enhancing functional properties like water/oil retention and adsorption capacities [108,109].

##### Ultrasonic Extraction for Enhanced Fiber Recovery

This gentler alternative uses sound waves to disrupt cells at lower temperatures. Optimal conditions (44 °C, 13 min, 38% amplitude, 1:40 sample-to-solvent ratio) yielded 52% fiber extraction from quinoa, improving hydrophilicity and creating unique microstructures. Ultrasonic methods reduce the processing time and solvent use while enhancing functional properties [109].

##### Pulsed Electric Field (PEF) Extraction for Fiber Isolation

PEF technology applies high-voltage pulses (8 kV/cm, 1 Hz, 20 µs pulse width) to permeabilize cell membranes, often combined with enzymatic treatments. Applied to peanut shells, this approach effectively reduced the protein and fat content while improving cholesterol/glucose adsorption and emulsification properties [30,108].

##### Subcritical Water Extraction (SWE): A Green Alternative

SWE, also referred to as pressurized hot water extraction, utilizes water maintained in its liquid state at elevated temperatures (between 100 and 374 °C) under pressure. This innovative extraction technique has been successfully applied for fiber extraction from various plant sources, including defatted coconut and wheat flour. The process typically employs a solvent-to-sample ratio of 1:30 at pH 6.5, with extraction conducted at 140 °C for 30 min. Research findings demonstrate that SWE significantly enhances the functional properties of extracted fibers, particularly improving their absorption capacity for glucose, cholesterol, and nitrite ions [110,111]. Compared to conventional methods, SWE offers several advantages: greater extraction efficiency, reduced processing time, and the use of water as an environmentally benign solvent. These benefits make SWE an attractive alternative for sustainable biomass valorization [108,110,111].

## 5. Functional and Biological Properties of Oilseed Components

### 5.1. Functional Characteristics of Extracted Proteins

Protein solubility, governed by the distribution of hydrophilic amino acids on protein surfaces, is generally low in oilseed proteins—soybean proteins show about 26% solubility at pH 7 [112] and sunflower proteins range from 20 to 30% at pH 4–6.5 [113]. Modern extraction techniques, like ultrasonic, enzymatic, high-pressure, and pulsed electric field (PEF) techniques can significantly enhance solubility; for instance, combining PEF extraction with a pH adjustment increased the soybean protein solubility from 26% to over 70% [112]. Low solubility and aggregation limit plant protein use in food applications, especially beverages [66]. The emulsification capacity and stability are key functional properties often limited in plant proteins but improved by advanced extraction methods. Rapeseed proteins (cruciferin and napin) show enhanced emulsifying properties when extracted with PEF extraction at a low pH, similarly to soybean proteins [112,114].

The foaming ability represents another critical functional property, characterized by the foaming capacity (FC) and foam stability (FS). These properties are particularly valuable in food products like creams and ice creams, where proteins contribute to texture and stability [115]. Sunflower proteins exhibit pH-dependent foaming with the maximum FC at pH 3 and FS at pH 5 [74]. While rapeseed 7S proteins show a poor foaming capacity, ongoing research explores how innovative extraction methods might enhance these properties [98].

Gelling properties vary widely: soy proteins form strong gels under acidic, high-temperature conditions, while sunflower proteins create weaker gels that improve with proteolysis but remain inferior to soy gels, likely due to the higher phenolic content [67,69].

### 5.2. Bioactive Potential of Oilseed Proteins

Plant proteins generally have a lower nutritional quality than animal proteins, measured by the Digestible Indispensable Amino Acid Score (DIAAS). Sunflower protein scores 84% but lacks lysine; soybean protein exceeds 75%, while rapeseed and canola fall below this value [116,117]. Enzymatic and PEF extraction can improve digestibility by modifying structures and reducing antinutrients [66,118]. Plant proteins are cholesterol-free and may reduce diabetes risk. Soy protein helps regulate blood glucose and lipids [119], while flax protein shows anti-diabetic effects [120]. Oilseed proteins contain bioactive peptides with a antihypertensive and antidiabetic activity, with rapeseed, sunflower, and sesame showing the highest ACE inhibition, though less than milk proteins [121]. Sunflower proteins additionally demonstrate antioxidant properties [118].

### 5.3. Health Benefits and Applications of Dietary Fibers

Dietary fibers provide numerous health benefits, including the prevention of constipation, obesity, diabetes, and cardiovascular diseases, along with potential cancer risk reduction. Soluble fibers particularly improve cholesterol levels, glucose tolerance, and insulin sensitivity, while serving as prebiotics for beneficial gut [105,122]. Fiber solubility, determined by structural features like carboxylic and sulfate groups, significantly influences bioactivity. Soluble fibers enhance viscosity, improving glycemic control and cholesterol reduction, while their gelling and emulsifying properties make them valuable food ingredients [106].

### 5.4. Health Benefits and Applications of Polyphenols

Polyphenols, abundant in oilseed cakes such as those from sunflower, rapeseed, flax, and sesame, are well-recognized for their potent antioxidant, anti-inflammatory, antidiabetic, and cardioprotective properties. These compounds include phenolic acids (e.g., chlorogenic, caffeic, ferulic acids), flavonoids, and tannins, whose profiles vary by oilseed type and extraction conditions [91,93].

#### 5.4.1. Bioactive Properties of Extracted Polyphenols

The antioxidant capacity of polyphenols is their most studied functional trait, primarily acting through radical scavenging and metal-chelating activities. Chlorogenic acid from sunflower cake and sinapic acid from rapeseed cake have shown a high DPPH and ABTS radical inhibition capacity [123]. These properties also enable improved oxidative stability in food products, particularly in oil-rich products, thereby preventing the development of off-flavors and extending shelf life [91,124]. Polyphenols also exhibit antimicrobial activity, particularly against Gram-positive bacteria and Gram-negative bacteria, with applications in active food packaging and shelf-life extension [125,126].

#### 5.4.2. Biological Potential of Oilseed-Derived Polyphenols

Oilseed polyphenols exert anti-inflammatory and antidiabetic effects by modulating key cellular pathways (e.g., NF-κB, MAPK, AMPK). Studies show that extracts from flaxseed and rapeseed cakes reduce markers of oxidative stress and improve glucose metabolism in cell and animal models [57].

In addition, anticancer activities have been reported, particularly for flavonoids and phenolic acids from sesame and sunflower meal, showing the inhibition of cancer cell proliferation and induction of apoptosis in vitro [28,57].

Their role as prebiotics is also emerging, with evidence that certain polyphenols modulate gut microbiota and promote the growth of beneficial bacteria [127].

#### 5.4.3. Applications in Functional Foods and Nutraceuticals

Due to their bioactivity, oilseed-derived polyphenol extracts are being incorporated into functional foods, dietary supplements, and beverages. They can also be used to enrich bakery products, dairy alternatives, or protein powders. Furthermore, their inclusion in edible films and coatings offers clean-label alternatives to synthetic additives [ref]. Encapsulation techniques (e.g., microencapsulation, nanoemulsions) are being explored to enhance polyphenol stability and bioavailability in food matrices [91,128].

## 6. Application and Valorization Strategies of Oilseed Cakes and Their Protein Isolates

The growing global population has intensified the demand for sustainable and economically viable protein sources. Oilseed cakes, currently underutilized as animal feed, represent a promising alternative due to their high protein content. These plant proteins offer nutritional benefits while addressing environmental concerns associated with traditional protein production [118].

Plant-based meat analogs predominantly utilize legume proteins, with soy protein being the most common choice despite its methionine deficiency and potential flavor impacts [67,129]. Oilseed proteins show potential in both meat alternatives and conventional meat products. While rapeseed protein-enriched sausages demonstrated superior taste and aroma compared to soy versions, they exhibited inferior texture and color characteristics [130]. These findings suggest that oilseed proteins require further refinement for optimal meat applications.

The growing market for plant-based milk alternatives has highlighted soy milk’s health benefits, including reduced risks of cancer and cardiovascular diseases [131,132]. Rapeseed and other oilseed proteins show promise for incorporation into protein drinks and dairy products, though their use is currently limited by negative impacts on sensory qualities when used in significant quantities [132,133].

Oilseed proteins address critical challenges in gluten-free baking, where they enhance both nutritional and functional properties. Research demonstrates that chia seed and rapeseed protein incorporation improves product quality, nutritional value, and shelf life in gluten-free bread formulations [134,135].

Oilseed protein isolates have emerged as valuable materials for developing nanocarriers that protect sensitive bioactive compounds. These systems include the following:

Nanoemulsions: Soy and rapeseed protein isolates effectively stabilize curcumin-loaded nanoemulsions (<200 nm), while also enhancing the stability and antioxidant properties of encapsulated β-carotene and vitamin E [131,136,137,138].

Nanogels: Cruciferin, the predominant canola protein (60–65% of total protein), demonstrates remarkable stability under harsh conditions (pH extremes, pepsin, 91 °C temperatures). This makes it particularly effective for protecting β-carotene during gastrointestinal transit and improving its bioavailability [139,140].

These advanced applications demonstrate how oilseed proteins can transcend traditional food uses to enable innovative solutions in both food and pharmaceutical sectors [80].

## 7. Conclusions

This review highlights the significant potential of oilseed cakes as sustainable sources of valuable nutrients and bioactive compounds for food and pharmaceutical applications. With global oilseed production continuously increasing, substantial quantities of these protein-rich (up to 56%) and fiber-containing (up to 66%) by-products remain underutilized. While traditionally used in animal feed and fertilizers, emerging extraction technologies—including ultrasound-assisted (yielding up to 96.64% phenolic compounds), enzymatic (82–83% protein recovery), and subcritical water extraction (enhancing functional properties at 140 °C)—enable the more efficient and environmentally friendly valorization of these resources. Applications range from improved gluten-free bakery products and plant-based meat alternatives to innovative nanoencapsulation systems for bioactive compounds, with soybean, rapeseed, sunflower, and flaxseed cakes each offering unique nutritional profiles and functional properties. Despite this progress, further development is required to overcome current limitations in process scalability, standardization, and regulatory acceptance. Challenges remain in optimizing extraction methods to balance yield, functionality, and cost-effectiveness while minimizing environmental impact. Future research and innovation should prioritize the implementation of green technologies, such as pulsed electric field (PEF) and high hydrostatic pressure (HHP) methods, at an industrial scale, developing integrated biorefinery approaches, and further exploring the health benefits of oilseed-derived peptides and fibers to enhance consumer acceptance and regulatory approval. These efforts will be key to unlocking the full potential of oilseed cakes within a circular and sustainable food system.

## Figures and Tables

**Table 1:** Proximate composition of oilseeds and oilseed cake *.

Oilseed and By-Product		Content (%)		References
	Oil	Protein	Fiber	
sunflower	28.0–60.0	10.0–25.5	16.0–55.0	[15,16,17,18,19]
soybean	7.3–24.7	14.0–47.0	5.6–31.9	[15,17,20]
rapeseed	31.2–47.5	18.6–20.1	12.8	[17]
flaxseed	30.0–42.2	18.3–30.0	20.0–35.0	[21,22]
rapeseed cake	1.1–31.3	14.0–45.0	5.5–19.5	[6,17,23,24]
soybean cake	0.5–8.9	25.7–52.4	3.0–6.0	[6,12,17,20,25,26]
sunflower cake	1.0–23.6	29.0–43.4	13.1–36.0	[18,19,27,28]
flaxseed cake	7.0–21.4	36.0–56.0	66.0	[24,29]

* Fiber values include structural carbohydrates (e.g., cellulose and hemicellulose) and lignin. Carbohydrates and lignin were not determined separately.

**Table 2:** Amino acid content of oilseeds cakes.

Amino Acid (%)	Type of Oilseed Cake			
	Sunflower	Soybean	Rapeseed	Flaxseed
Isoleucine (Ile)	1.20–4.20	2.10–5.20	1.19–3.80	2.14–2.17 *
Leucine (Leu)	2.00–6.90	3.35–7.80	2.09–6.30	
Lysine (Lys)	0.86–3.5	2.02–2.66	1.62–5.40	0.25–1.40
Methionine (Met)	0.51–2.20	0.25–1.40	0.59–1.70	0.38–0.51
Cysteine (Cys)	0.78–1.80	0–0.57	0.72	0.36
Phenylalanine (Phe)	0.70–5.10	1.73–5.50	1.17–3.80	0.94–1.79
Tyrosine (Tyr)	0.61–1.40	1.15–1.42	n.d **	0.57–0.83
Threonine (Thr)	1.20–3.40	1.01–3.80	1.34–3.80	0.65–1.46
Tryptophan (Trp)	0.80–1.40	0.57–1.15	0.80–1.30	0.46
Valine (Val)	1.50–5.80	1.44–5.20	1.56–5.20	0.96–2.41
Arginine (Arg)	1.83–9.10	1.41–1.79	1.77–6.40	2.16–4.76
Histidine (His)	0.70–2.80	0.78–2.40	0.78–2.60	0.48–0.53
Alanine (Ala)	0.60–2.04	1.21–2.15	1.32–1.50	0.77
Aspartic acid(Asp)	1.83–9.10	4.71	2.18	2.23
Glutamic acid (Glu)	0.13–4.22	7.85	4.70	2.96
Glycine (Gly)	1.48–5.60	1.15–4.50	1.56–4.90	1.46–2.23
Proline (Pro)	1.45–2.21	2.13–2.43	1.75	0.66–1.82
Serine (Ser)	1.11–1.92	1.27–2.60	1.25–1.5	0.84–1.82
References	[30,31,32]	[32,33,34]	[32,35,36]	[34,37]

* sum of isoleucine (Ile) and leucine (Leu). ** not detected.

**Table 3:** Protein fractions, micronutrients, bioactive, and antinutritional compounds in major oilseed cakes.

Type of Oilseed Cake	Protein Fractions	Minerals	Vitamins	Bioactive Compounds	Antinutritional Factors
Sunflower	40.0–90.0% globulins (helianthinin), 10–30% albumin (2S albumin) [19,38]	5.0–6.0% minerals: K(1.5%), P (1.2%), Ca (0.7%), Mg (0.4%), Na (0.03%) Tl, Cu, Zn, Cr, Mn [39,40]	nicotinic acid, thiamine, pantothenic acid, riboflavin [16,17,24,25,39]	1.0–4.0% phenolic compounds (chlorogenic (70.0%), caffeic, and quinic acids) [19,27,41]	chlorogenic acid [27]
Soybean	90.0% globulins (36 to 53% glycine, 30 to 46% β-conglycinin) 10.0% albumins) [17,42]	5.9–6.0% minerals: K (2.1%), P (0.7%), Ca (0.3%), Mg (0.2%), Na (0.02%), Fe, Zn, Mn, Cu [12,15,40]	thiamine, riboflavin, niacin, folic acid, [15,26]	flavonoids, isoflavones (genistein, daidzein), lignans, and saponins [20,26,43]	trypsin inhibitors, lectins, goitrogens [42]
Rapeseed	55.0–85.0% globulin (cruciferins) 15.0–45.0% albumin (napins) [44,45]	6.5–6.8% minerals: K(0.8%), P (0.6%) (65% p as phytate), Ca(0.4%), Mg (0.259, Na (0.02%), Se, Fe, Mn [40,46]	choline (0.7%), niacin (0.02%), biotin, folic acid, niacin, [17,25]	polyphenols (sinapic acid), [45,47]	glucosinolates (progoitrin, gluconapin, glucobrassicanapin) [17,46,48]
Flaxseed	45.0–80.0% globulins (linins), 6–17,7% albumins (colinis) [17,49,50,51]	3.9–5.4% minerals: Na (1.0%), P (0.9%), Ca (0.3%), Mg (0.003%), K, Zn, Mn, Cu [37,40,48,52]	thiamine, riboflavin, pyridoxine, niacin folic acid [49,50,51,53]	phenolic acid (ellagic, ferulic, guercetic acids), flavonoids, and lignans (SDG) (0.2–2.4%) [54,55]	cyanogenic glycosides (linustatin, neolinustatin, linumarin), phytic acid (~1.5%), and tannins [53]

**Table 4:** Bioactive compounds of oilseeds.

Oilseed	Bioactive Compounds	References
sunflower	p-coumaric acid, chlorogenic acid, caffeic acid, syringic, vanillic, gallic acid, vanillic acids, catechin, epicatechin	[93,94]
soybean	p-coumaric acid, ferulic acid, hesperidin, rutin, sinapic acid, syringic acid, gallic acid, hydroxybenzoic acid, vanillic acid	[91,95,96]
rapeseed (canola)	gallic acid, p-coumaric, caffeic acid, ferulic acid, epicatehin, sinapic, esters, and glycosides of phenolic acid	[3,91,95]
flaxseed	tannic acid, p-coumaric, ferulic acid, p-hydroxybenzoic acid, lignans, sinapic, lignin	[91,95,96]

## Data Availability

No new data were created or analyzed in this study.
